# Dryland irrigation increases accumulation rates of pedogenic carbonate and releases soil abiotic CO_2_

**DOI:** 10.1038/s41598-021-04226-3

**Published:** 2022-01-10

**Authors:** Anna C. Ortiz, Lixin Jin, Nives Ogrinc, Jason Kaye, Bor Krajnc, Lin Ma

**Affiliations:** 1 Department of Geological Sciences, University of Texas at El Paso, 500 W. University Ave, El Paso, TX 79968 USA; 2 Department of Environmental Sciences, Jožef Stefan Institute, Ljubljana, Slovenia; 3 Department of Ecosystem Science and Management, Pennsylvania State University, University Park, PA 16802 USA

**Keywords:** Carbon cycle, Element cycles

## Abstract

Agricultural fields in drylands are challenged globally by limited freshwater resources for irrigation and also by elevated soil salinity and sodicity. It is well known that pedogenic carbonate is less soluble than evaporate salts and commonly forms in natural drylands. However, few studies have evaluated how irrigation loads dissolved calcium and bicarbonate to agricultural fields, accelerating formation rates of secondary calcite and simultaneously releasing abiotic CO_2_ to the atmosphere. This study reports one of the first geochemical and isotopic studies of such “anthropogenic” pedogenic carbonates and CO_2_ from irrigated drylands of southwestern United States. A pecan orchard and an alfalfa field, where flood-irrigation using the Rio Grande river is a common practice, were compared to a nearby natural dryland site. Strontium and carbon isotope ratios show that bulk pedogenic carbonates in irrigated soils at the pecan orchard primarily formed due to flood-irrigation, and that approximately 20–50% of soil CO_2_ in these irrigated soils is calcite-derived abiotic CO_2_ instead of soil-respired or atmospheric origins. Multiple variables that control the salt buildup in this region are identified and impact the crop production and soil sustainability regionally and globally. Irrigation intensity and water chemistry (irrigation water quantity and quality) dictate salt loading, and soil texture governs water infiltration and salt leaching. In the study area, agricultural soils have accumulated up to 10 wt% of calcite after just about 100 years of cultivation. These rates will likely increase in the future due to the combined effects of climate variability (reduced rainfall and more intense evaporation), use of more brackish groundwater for irrigation, and reduced porosity in soils. The enhanced accumulation rates of pedogenic carbonate are accompanied by release of large amounts of abiotic CO_2_ from irrigated drylands to atmosphere. Extensive field studies and modelling approaches are needed to further quantify these effluxes at local, regional and global scales.

## Introduction

Irrigated agriculture is expanding in drylands to produce crops that meet rising food demands and support local economy^1,2^. However, soil salinization due to continuous irrigation is a global problem that affects agricultural soils from Asia, Europe, North America, and Australia, covering approximately 20% of irrigated area around the world^3–5^. Soils have been converted to managed agricultural fields along the Rio Grande valley in western Texas and southern New Mexico for more than 100 years^6^. This region is a typical dryland system with mean annual precipitation at ~ 16–25 cm and annual potential evapotranspiration at ~ 194 cm^7,8^. During growing seasons, large areas of cropland are inundated by diverting Rio Grande river water through canals and by pumping groundwater from deep aquifers. Such flood irrigation is not a water-conservative method and has led to high evaporative water loss especially during hot and dry summers, salt accumulation, and a decrease in soil permeability, quality and productivity^8–14^.

**Figure 1:**
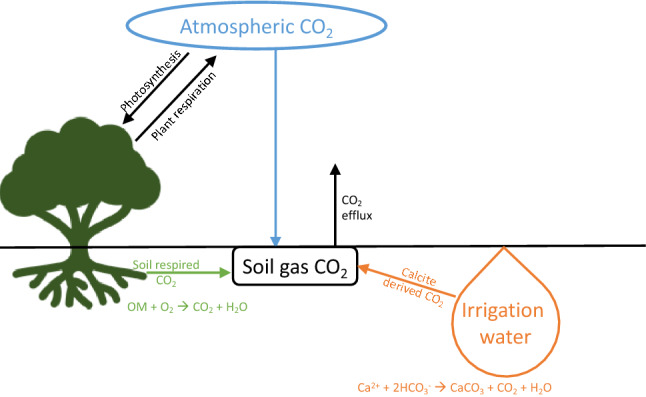
A simplified conceptual model to highlight major CO_2_ fluxes and calcite formation pathways in the agricultural fields. Soil CO_2_ is contributed by atmospheric, soil respired and calcite derived CO_2_.

Soil CO_2_ is produced naturally by decomposition of organic matter, root respiration and microbial respiration; in dryland environments, an additional abiotic source is pedogenic carbonate precipitation (Fig. 1). Indeed, irrigation practices deliver large amounts of dissolved Ca^2+^ and HCO_3_^−^ to soils, leading to precipitation of pedogenic carbonate even before water-soluble evaporite salts such as gypsum and halite^12,14–16^. To date, however, a limited number of studies have identified the accelerated accumulation rates of pedogenic carbonate (secondary calcite) in such agricultural settings^17–24^, and only a few have attempted to measure the release of CO_2_ (referred to as abiotic CO_2_) through carbonate precipitation^25–30^:

1 $${\text{Ca}}^{2 + }_{{({\text{aq}})}} + 2{\text{HCO}}_{{3({\text{aq}})}}^{ - } \leftrightarrow {\text{CaCO}}_{{3({\text{s}})}} + {\text{CO}}_{{2({\text{g}})}} + {\text{H}}_{2} {\text{O}}_{{({\text{aq}})}}$$

It is essential to identify and quantify such abiotic CO_2_, investigate the controls on its efflux from soils to atmosphere, and upscale the interactive effects of climate change and irrigation expansion on C cycling in drylands.

Here we report a case study to reveal the linkages among irrigation, salt loading and CO_2_ dynamics from dryland areas, in the Rio Grande valley near El Paso, Texas. Here, soils are developed on ancestral Rio Grande floodplain sediments and natural pedogenic carbonate has formed naturally for thousands of years^31,32^. The study sites include two agricultural sites and one natural site (Fig. S1), and both farms have been intensively cultivated and flood irrigated for the last 100 years^6^. We hypothesize that flood irrigation has produced younger pedogenic carbonate of different carbon and strontium isotopic signatures as compared to the natural counterparts, and detectable abiotic CO_2_. This study builds on previous investigation on sources and controls of soil salinity in agricultural soils^12–14^, and collects new C and Sr isotope data to determine the relative contribution of irrigation-induced reaction (1) to the overall soil CO_2_ and bulk soil pedogenic carbonate.

## Methods and materials

### Study sites

Our study focused on one natural site and two agricultural sites along the Rio Grande valley in southern New Mexico and western Texas, USA (Fig. S1A). Soils in all sites are developed on alluvial sediments of the ancient Rio Grande river^14^. The two agricultural sites are located in the El Paso County of western Texas: a pecan orchard in Tornillo, TX and an alfalfa field in El Paso, TX. The pecan field has been cultivated for pecans for approximately last 40 years, and for cotton for 60 years prior (Fig. S1B, S1C). The alfalfa field has only been cultivated for alfalfa. With low annual mean precipitation in the American southwest, these agricultural fields are flood irrigated, primarily with Rio Grande river water. The pecan orchard is typically irrigated from April to October during the growing season, once every two to three weeks with approximately 10 to 12 irrigation events or on average 1.5 m of water annually. When river water is insufficient, the pecan orchard would be irrigated using local groundwaters, while the alfalfa field is left fallow. In addition, the pecan orchard is fertilized and also amended by sulfur and gypsum pellets to lower soil sodicity^14^. The alfalfa site is less managed and has no history of soil amendments or fertilization. A natural site was selected for comparison, in the Chihuahuan desert scrub rangeland outside of Fabens, Texas, that has never been cultivated or irrigated (Fig. S1D).

### Soil and dust sample collection and characterization

These three sites have been previously studied for soil texture, salinity and sodicity^12–14,33^. Soil salinity is measured as electrical conductivity (EC) and sodicity as sodium adsorption ratio (SAR), the ratio of sodium concentration over the square root of the sum of calcium and magnesium concentrations in a soil slurry. Soil sodicity controls clay behavior, soil structure and soil permeability. Archived soil, dust and water samples were used for this study and their collection is briefly described here. At the pecan orchard, two soil cores were collected at ~ 10 cm increment resolution, with contrasting texture: Pecan_Fine and Pecan_Coarse, to the depths of 250 cm and 300 cm respectively. Pecan_Fine soils have a layer of clayey soils around 100–150 cm and have much higher soil salinity and sodicity than the sandy Pecan_Coarse soils. These two soil profiles are less than 20 m apart and undergo the same soil amendments and irrigation schedule. However, the Pecan_Fine and Pecan_Coarse soils are characterized by visually distinct pecan growth, mainly due to different amounts of salt buildup. A 60-cm deep soil core was previously collected and characterized in an alfalfa field (as alfalfa)^12^ and as Alfalfa_Fine_D^14^ in El Paso, Texas. These soils are silty clay loam alluvium of Harkey-age (NRCS Custom Soil Report). As a natural site for comparison, a 110-cm deep core was augured at Fabens, Texas and studied for soil salinity and dust deposition^14^.

Dust, as a potential source of Ca in pedogenic carbonates, was sampled at both the pecan orchard site and the natural Fabens site using passive dust traps 150 cm above ground^34–36^. Both dust traps were maintained for one year (2015–2016) and a single sample was collected from each representing a composite of dry and wet deposition and by washing the beads.

Soil carbon (SC) and organic carbon (SOC) contents were quantified using a LECO SC632 analyzer at the Low-Temperature Geochemistry Laboratory of the University of Texas at El Paso (UTEP). About 0.2 g of ground bulk soil samples was weighed, mixed with combustion catalyst, and combusted in the LECO furnace at 1450 °C. The moisture was then removed from the resultant gases and CO_2_ was quantified to calculate SC. Two standards of different carbon contents and different weights were used for calibration. A soil reference material is run with the samples as checks and the measured values are within 0.1 wt% of reference values. Another aliquot of bulk soils was treated by 1:1 HCl to remove carbonate (also known as soil inorganic carbon; SIC) and then dried in the oven at 60 °C. The carbon content measured on these acid-leached soils was considered as SOC, and SIC was calculated as the difference between SC and SOC. Weight percent of calcite was then computed from SIC based on calcite stoichiometry (CaCO_3_). Two pure calcite samples were run with the same procedure and measured C contents were lower than 0.02% ensuring complete release of C during acidification.

The C isotopes of organic matter (*δ*^13^C_SOC_) and carbonate (*δ*^13^C_CaCO3_) in the selected soil samples were analyzed on a continuous-flow isotope ratio mass spectrometer (IRMS; Finnigan Delta PlusXL) at the University of Arizona. Precision for *δ*^13^C_SOC_ was ± 0.1‰ or better (1σ). In order to measure C isotope composition of the carbonate minerals (*δ*^13^C_CaCO3_), soil samples were reacted with dehydrated H_3_PO_4_ under vacuum at 70 °C. The released CO_2_ was then measured by an IRMS (Finnigan MAT 252). For these carbon isotope measurements, precision is better than ± 0.08‰ (1σ).

The sequential extraction of water leachable and acid leachable fractions of the soils were conducted for the pecan, alfalfa, and natural Fabens sites as well as two dusts from Fabens and pecan field^14^. The water fraction was used to dissolve evaporite salts, such as CaCl_2_, NaCl, CaSO_4_ and Na_2_SO_4_, and directly linked to irrigation through soil salinity. Specifically, 10 g of a soil sample and 1 g of dust sample was weighed into a centrifuge tube, with 30 mL of de-ionized water (18.2 MΩ). The slurry was shaken for 15 min on a shaker and centrifuged at 3500 rpm for ten minutes. The supernatant was passed through with a 0.45 μm filter and weighed. Exchangeable cations were extracted with 25 mL of 0.1 M BaCl_2_–0.1 M NH_4_Cl from soils and dusts between water soluble and acid leachable fractions, to remove cations adsorbed to clays and Fe or Al oxyhydroxides. The acid leachable fraction dissolved carbonate minerals such as secondary calcite and amorphous Fe or Al oxides if present. Specifically, 20 mL of 1 M or 2 M acetic acid was added onto soil residue from the CEC fraction, depending on the soil inorganic carbon concentrations. The mixture was shaken for 6 h and centrifuged at 2500 rpm for 20 min and the supernatant filtered with 0.45 μm paper filter. The soil residue was washed again with 3 mL of 1 M or 2 M acetic acid to ensure the carbonate fraction was completely dissolved and collected. Two aliquots of acetic acid leachates were combined, dried and dissolved in 2% HNO_3_. Leachates from these two sequential extractions were analyzed for major elements to evaluate soil salinity and sodicity (water leachable fraction) and for abundance of pedogenic carbonate (based on Ca in acid leachable fraction)^14^. These leachates are analyzed for Sr isotopes as discussed below.

### Water sample collection and characterization

Nested lysimeters were placed at 15, 30, 60, and 120 cm depths for both pecan sites, Pecan_Fine and Pecan_Coarse. Rio Grande and groundwater irrigation samples, as well as soil waters, were only collected from the pecan orchard. Lysimeter waters were collected and filtered using 0.45 µm membrane filters. The pH, EC, alkalinity (equivalent of dissolved inorganic carbon, DIC at this pH), concentrations of major ions, and saturation indexes with respect to calcite were previously reported^14^. Irrigation water samples collected at the study sites included Rio Grande surface waters (IRW_RG), and local groundwaters (IRW_GW), where the groundwater is much more concentrated than river water, with higher Na^+^ and Ca^2+^ concentrations and thus higher potential to increase soil salinity and sodicity^14^. For these water samples, new C and Sr isotope data were collected and reported (Appendix Table 2). The C isotopes of dissolved inorganic carbon (with HCO_3_^−^ as the dominant DIC species, average pH = 7.74; *δ*^13^C_DIC_) was measured on a continuous-flow gas-ratio mass spectrometer (ThermoQuest Finnigan Delta PlusXL) coupled with a Gasbench automated sampler at the Environmental Stable Isotope Laboratory at the University of Arizona. The samples were acidified with phosphoric acid at room temperature in Exetainer vials previously flushed with He gas. The precision in *δ*^13^C_DIC_ is better than ± 0.3‰ (1σ).

Soil leachates and water samples were analyzed for Sr isotope analysis. Twenty-five milliliters of the full-strength filtered waters were dried in 30 mL Teflon beakers, then dissolved in 0.5 mL of 3.5 N HNO_3_^−^ before the Sr elution sequence for isotopic analysis. Strontium purification was conducted in a Class 100/1000 clean room-laminar air fume hood with Eichron® Sr 100–150 µm resin in 1.5 mL Teflon columns at UTEP. Two elution Sr purification sequences were conducted to attain an evaporable aliquot for analysis. Seven rinses with 3.5 N HNO_3_ acid were performed in each sequence then an eighth rinse with 0.05 N HNO_3_ to yield the purified sample. All purified samples were then analyzed for ^87^Sr/^86^Sr isotopes on the multi-collector inductively coupled plasma mass spectrometry (MC-ICP-MS) at UTEP using standard-sample bracketing method^37^. The Sr isotope bracketing standard SRM 987 yielded average ^87^Sr/^86^Sr ratios of 0.710235 ± 0.000005 (2SE, n = 32). For quality control purposes, USGS BCR2 rock standards were treated as bulk samples, with measured average ^87^Sr/^86^Sr ratios of 0.70502 ± 0.00001 (2SE, n = 9) that were within the values reported in literature (0.70502)^38^. Sr blanks were negligible pico-gram scales (~ 80 pg). For soil leachates, the uncertainty is typically within 0.0001 (2SE) due to low amounts of Sr used for analysis except for several soils on the water soluble fraction. For water samples, ^87^Sr/^86^Sr has a much lower uncertainty at 0.00002 (2SE, n = 7) as a result of high amounts of Sr used for analysis.

### Soil gas sample collection and analyses

Two nests of soil gas tubes were installed at the Pecan_Fine and Pecan_Coarse sites at four depths (15, 30, 60 and 120 cm) and sampled following a modified USGS protocol^39,40^. Soil gas samples were collected using 60-mL gas-tight plastic syringes and needles after purging two tube volumes to clear the sampler tube. Gas samples were immediately transferred to pre-evacuated 15-mL LETCO^®^ glass vials. Additional atmospheric gas samples for pCO_2_ and δ^13^C_CO2_ were taken as local atmospheric endmembers, as well as for quality control. Gas samples were collected prior to irrigation and also one week after each flooding event, when the pecan field was dry enough to be accessible for soil gas sampling.

Soil gas CO_2_ concentrations (pCO_2_) were measured by a LiCOR 7000 gas analyzer, calibrated with CO_2_ standards with concentrations of 970 and 10,300 ppmv at Pennsylvania State University. Check standards were analyzed along with unknown samples and the precision in CO_2_ concentrations is better than ± 5% of the reference values. The isotopic composition of soil CO_2_ was determined using a Europa 20–20 continuous flow Isotope Ratio Mass Spectrometer (IRMS) with an ANCA-TG preparation module for trace gas samples at the Jozef Stefan Institute in Slovenia. Gas samples were flushed with He across two chemical traps that removed water and then trapped the CO_2_. Precision in *δ*^13^C_CO2_ is better than ± 0.1‰ (1σ), based on repeated internal standards.

## Results and discussion

### Controls on C isotope compositions of soil gas CO_2_ of an intensively irrigated Pecan field

The pCO_2_ of soil gases within the pecan orchard increases with depth (Appendix Table 1; Fig. S2A), characterized by a typical diffusion profile where CO_2_ is emitted to atmosphere^39,41^. Up to 74,000 ppm, or almost 200 times of the atmospheric CO_2_ level are observed in deep soils, below 60 cm depth. These concentrations are even higher than some natural vegetated systems^39,40,42^, suggesting enhanced soil respiration, root respiration and microbial activities as expected from soil cultivation and growth of pecan trees. The soil organic carbon (SOC) contents in both soil profiles are high near ground surface, at ~ 1.5 wt%, and decreases sharply with depth (Fig. S3A).

**Figure 2:**
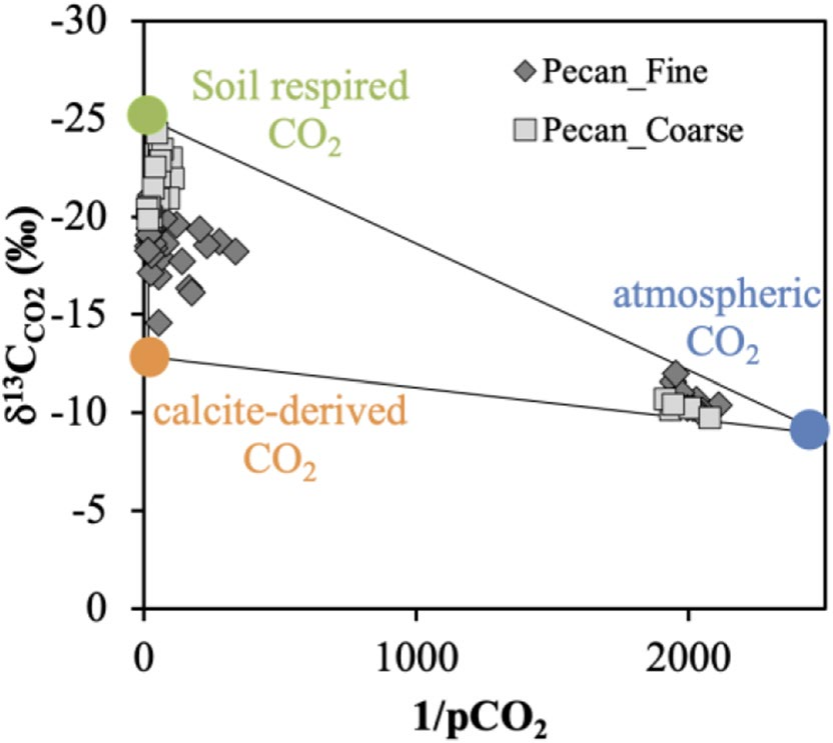
Keeling plot shows soil CO_2_ is contributed by three endmember mixing (atmospheric, soil respired and calcite-derived abiotic) in two soil profiles. Pecan_Fine has finer soil texture and higher calcite content than Pecan_Coarse, and soil CO_2_ in Pecan_Fine receives more calcite-derived abiotic CO_2_ contribution. Pecan_Coarse soils support larger trees and so, produce more soil respired CO_2_. The atmospheric *δ*^13^C_CO2_ is − 8.9‰^43^.

The isotope ratio of soil CO_2_ (*δ*^13^C_CO2_) varied dramatically with depth and between Pecan_Fine and Pecan_Coarse sites (Fig. S2B). The Keeling plot revealed two clusters in all soil gas samples, with at least three endmembers of different C isotope compositions (Fig. 2). Soil gases from shallow depths are closer to the atmospheric endmember^43^ (blue circle). Soil respired CO_2_ endmember (green circle in Fig. 2) has the same carbon isotope compositions as soil organic carbon (*δ*^13^C_SOC_) between − 21.5 and − 24.5‰ (Fig. S3B). The third possible CO_2_ endmember (calcite-derived, abiotic) is discussed below (Orange circle in Fig. 2).

**Figure 3:**
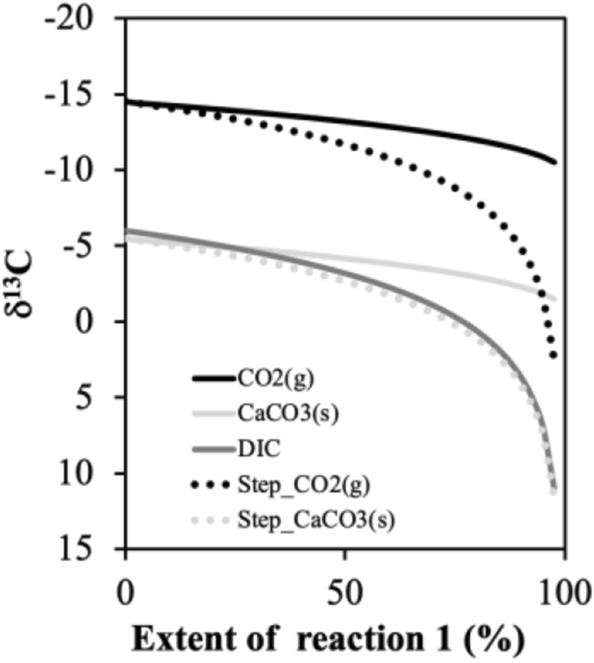
Evolution in *δ*^13^C values of CaCO_3_ and CO_2_ is modelled assuming Rayleigh fractionation, as DIC and Ca^2+^ in the irrigation water slowly precipitate out according to chemical reaction (1) with initial δ^13^C_DIC_ at − 6‰. Isotopic composition is plotted as dotted lines for each step or as solid lines cumulatively, for each species (black for CO_2_ (g), grey for CaCO_3_(s) and dark grey for DIC). When all DIC is converted to calcite and CO_2_, extent of the reaction moves from 0 to 100%, and the carbon isotopes of these endmembers are derived (*δ*^13^C_CaCO3_ = − 1.75‰; *δ*^13^C_CO2_ = − 12.85‰).

The δ^13^C_DIC_ of the Rio Grande water samples used for irrigation ranges from − 10.4 to − 6.3‰, on average − 7.3‰ (n = 6); while groundwaters used for irrigation have more negative δ^13^C_DIC_ ratios, averaging at − 11.3‰ (n = 2) (Appendix Table 2). The *δ*^13^C of DIC, CaCO_3_ and CO_2_ constituents can be modeled using a Rayleigh fractionation when irrigation water or soil water evaporates, and CaCO_3_ and CO_2_ form according to Reaction (1). This process assumes an open system where DIC in soil water is an isotopically homogenous and finite reservoir, and the reaction products (CaCO_3_ and CO_2_) that are instantaneously formed are always at isotopic equilibrium with remaining reactant (DIC) and are then continuously removed and isolated from the liquid phase (DIC)^44^. The fractionation factors between calcite and DIC (namely HCO_3_^−^ at neutral pH, as observed in the irrigation water) and between CO_2_ and DIC are assumed to be constant, and at 20 °C, the average soil temperature at the field sites, ε^13^C_CO2(g)-HCO3_ is − 8.5‰ and ε^13^C_CaCO3(s)-HCO3_ is 2.6‰^44^. We used an initial DIC of 4 mM, and modelled the evolution of carbon isotopes with each step of 0.1 mM DIC (Fig. 3). The continuous production of calcite and CO_2_ leads to higher *δ*^13^C_DIC_ in the residual soil water as reaction proceeds (Fig. 3). As a result, the solid and gas phases from each precipitation step (dashed lines) become more enriched in *δ*^13^C.

2 $$\updelta ^{13} {\text{C}}_{{{\text{CO}}2({\text{t}} + 1)}} =\updelta ^{13} {\text{C}}_{{{\text{DIC}}({\text{t}})}} - 8.5\textperthousand;\quad\updelta ^{13} {\text{C}}_{{{\text{CaCO}}3({\text{t}} + 1)}} =\updelta ^{13} {\text{C}}_{{{\text{DIC}}({\text{t}})}} + 2.6\textperthousand$$

3 $$\updelta ^{13} {\text{C}}_{{{\text{DIC}}({\text{t}} + 1)}} *{\text{DIC}}_{{({\text{t}} + 1)}} =\updelta ^{13} {\text{C}}_{{{\text{DIC}}({\text{t}})}} *{\text{DIC}}_{{({\text{t}})}} +\updelta ^{13} {\text{C}}_{{{\text{CO}}2({\text{t}} + 1)}} *\left[ {{\text{CO}}_{2} } \right] +\updelta ^{13} {\text{C}}_{{{\text{CaCO}}3({\text{t}} + 1)}} *\left[ {{\text{CaCO}}_{3} } \right]$$

According to Reaction (1), the amount of CO_2_ or CaCO_3_ formed during each step:

4 $$\left[ {{\text{CO}}_{2} } \right] = \left[ {{\text{CaCO}}_{3} } \right] = \raise.5ex\hbox{$\scriptstyle 1$}\kern-.1em/ \kern-.15em\lower.25ex\hbox{$\scriptstyle 2$} \left( {{\text{DIC}}_{{({\text{t}} + 1)}} - {\text{DIC}}_{{({\text{t}})}} } \right)$$

The Ca^2+^/DIC molar ratios are variable in the Rio Grande river water, and much greater than 2 in the local groundwater (Appendix Table 2)^14^; overall there should be a Ca^2+^-surplus after all DIC precipitates out of soil water according to Reaction (1). This is consistent with the presence of water-soluble and Ca-bearing evaporite salts in these soils^12,14^.

When 100% of DIC reacts from the consequent drying of this irrigation event, mass balance considerations require that the isotope content of the total accumulated gas CO_2_ and solid CaCO_3_ approaches the initial water DIC:

5 $$\updelta ^{13} {\text{C}}_{{{\text{DIC}}}} *{\text{DIC}} =\updelta ^{13} {\text{C}}_{{{\text{CO}}2}} *\left[ {{\text{CO}}_{2} } \right] +\updelta ^{13} {\text{C}}_{{{\text{CaCO}}3}} *\left[ {{\text{CaCO}}_{3} } \right]$$

6 $${\text{While}}\;\left[ {{\text{CO}}_{2} } \right] = \left[ {{\text{CaCO}}_{3} } \right] = \raise.5ex\hbox{$\scriptstyle 1$}\kern-.1em/ \kern-.15em\lower.25ex\hbox{$\scriptstyle 2$} \;{\text{DIC}}$$

Rearranging Eq. (2) the difference in C isotopes between CaCO_3_ and CO_2_ is always:

7 $$\updelta ^{13} {\text{C}}_{{{\text{CaCO}}3}} -\updelta ^{13} {\text{C}}_{{{\text{CO}}2}} = 11.1\textperthousand$$

Thus Eq. (5) becomes: δ^13^C_DIC_ * DIC = ½ DIC * (2δ^13^C_CaCO3_ − 11.1‰) and with δ^13^C_DIC_ = − 7.3‰, δ^13^C_CaCO3_ = (− 14.6 + 11.1)/2 = − 1.75‰; δ^13^C_CO2_ = − 12.85‰.

This is consistent with the model results in Fig. 3: the *δ*^13^C_CaCO3_ of all calcite that precipitates out is estimated at − 1.75‰ and the *δ*^13^C_CO2_ of all CO_2_ is at − 12.85‰. This inclusion of the calcite-derived abiotic CO_2_ endmember explains the deviation of soil gas CO_2_ cluster from the atmosphere-soil respiration mixing curve. Our study has observed a detectable soil CO_2_ from the calcite precipitation, loaded and driven by irrigation.

For deep soil gases (60 cm or deeper) without the influence of atmospheric CO_2_, a two-component mixing system (A = irrigation-derived abiotic CO_2_, B = soil respired CO_2_) can be used to calculate the fraction of CO_2_ in the bulk soil gas (mix) from source A:

8 $$X_{A} = \frac{{R_{mix} - R_{B} }}{{R_{A} - R_{B} }}$$

where R_A_ = *δ*^13^C_CO2_ = − 12.85‰; R_B_ = *δ*^13^C_CO2_ = − 24.5‰; and R_mix_ = *δ*^13^C_CO2_ = − 18.8 ± 1.5‰ (n = 36, at Pecan_Fine) or − 21.7 ± 1.4‰ (n = 14, at Pecan_Coarse). Solving for X_A_, the irrigation-derived abiotic CO_2_ at the clayey Pecan_Fine site is shown to contribute ~ 49% (range: 37% to 62%) of bulk CO_2_ at deeper soils where atmospheric input is negligible, much higher than at the sandy Pecan_Coarse site (average ~ 24%, range: 12 to 36%). This difference is attributed to contrasting soil texture and water dynamics. First, clayey soils at the Pecan_Fine site reduced water infiltration and leaching, and promoted higher salt buildup; here, the soil salinity exceeded tolerance levels of pecan trees and stunted their growth. Thus less soil respired CO_2_ was produced at the Pecan_Fine soils than Pecan_Coarse soils. Second, salt buildup was more pronounced at the Pecan_Fine site, including secondary calcite (Fig. 5A, see below). Therefore, more abiotic CO_2_ is expected to be released from calcite precipitation at the Pecan_Fine site.

Independently, the relative importance of these two sources can be evaluated by a mass balance calculation. Alkalinity (a proxy of DIC at the measured pH 7.2–8.1) of the irrigation water is at 3.6 ± 0.1 meq/L (n = 5) for the Rio Grande and 6.1 ± 1.0 meq/L (n = 2) for the groundwater (Appendix Table 2). On average, 5 inches of water (12.7 cm) are used for an irrigation event, equivalent of 127 L/m^2^. Thus, the calcite or abiotic CO_2_ produced through Reaction (1) will be half of the DIC loaded by the irrigation water (e.g., Rio Grande) or 0.23 mol over 1 m by 1 m ground surface (S = 1 m^2^). If this abiotic CO_2_ is evenly distributed over a 2-m vadose zone, the deepest water level observed locally, this will increase soil gas pCO_2_ by:

9 $${\text{pCO}}_{2} = {\text{nRT/V}}$$

where n = 0.23 mol, R is gas constant, and T is the soil temperature (20 °C, or 293.15 K). V is the volume of soil gas in this 1 m by 1 m by 2 m deep soil profile, and we assume 30% of porosity^12^ and half of the pore space is occupied by water:

$$\begin{aligned} & {\text{V}} = 2\;{\text{m}}^{3} *30\% *50\% = 0.3\;{\text{m}}^{3} \\ & {\text{pCO}}_{2} = 0.23*\left( {8.20578*10^{ - 5} } \right)*293.15{/}0.3 = 18*10^{ - 3} \;{\text{atm}}\;{\text{or}}\;1.8\% \\ \end{aligned}$$

These concentrations might vary with porosity, temperature, and other variables, but they are in the same order of magnitude of bulk pCO_2_ measured in soil gases, providing another line of evidence that abiotic CO_2_ contribution is detectable through both CO_2_ concentrations and carbon isotope ratios. If such abiotic CO_2_ is released to the atmosphere over three weeks until the next irrigation event, the average CO_2_ efflux can be estimated:

$${\text{F}}_{{{\text{CO}}2}} = {\text{n/t/S}} = 0.23\;{\text{mole/}}3\;{\text{weeks/}}1\;{\text{m}}^{2} = 0.13\;\upmu {\text{mol}}\;{\text{C}}\;{\text{m}}^{ - 2} \;{\text{s}}^{ - 1} \;{\text{or}}\;48\;{\text{gC}}\;{\text{m}}^{ - 2} \;{\text{yr}}^{ - 1}$$

However, this efflux is not constant, and its temporal fluctuation is controlled by CO_2_ production and equally importantly, by gas transport. For example, the irrigation water floods the field and soil gas will be pushed out by water, emitting a large CO_2_ flux.

### Separating naturally formed versus anthropogenic-driven pedogenic carbonates

#### Sr isotope systematics to trace Ca sources

**Figure 4:**
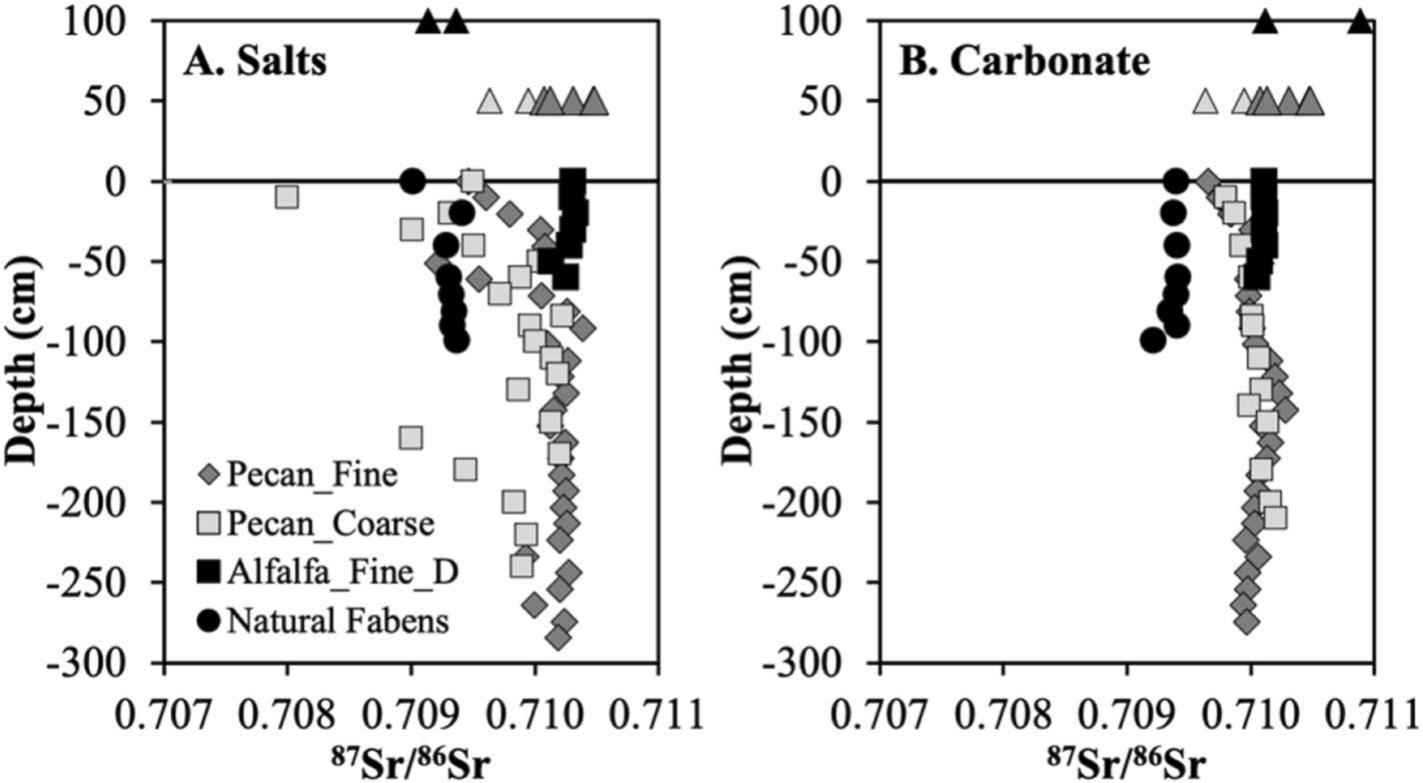
Depth profiles of ^87^Sr/^86^Sr ratios in evaporite salts (**A**, water leachable fraction) and pedogenic carbonate (**B**, acid leachable fraction) at three agricultural sites (Pecan_Fine and Pecan_Coarse at a pecan orchard, Alfalfa_Fine_D at an alfalfa field) and the natural Fabens site nearby. Also shown are Sr isotope compositions of two dust samples (black triangles, plotted at depths of 100 cm) and irrigation waters (plotted at 50 cm; light triangles for groundwater, IRW_GW and darker triangles for the Rio Grande, IRW_RG).

Sr isotope data were collected on five soil profiles (two on the alfalfa site, two on the pecan site, and one on natural Fabens site) on water leachable (evaporite salts) and acid leachable (mainly pedogenic carbonate) fractions (Appendix Table 3). Soils at the natural Fabens site have a narrow range of ^87^Sr/^86^Sr ratios in the evaporate salts and pedogenic carbonate fractions, between 0.70921 and 0.70941, and are also similar to dusts in their salt fractions (Fig. 4). However, the pedogenic carbonate of dusts had different Sr isotopes from their salt fractions. Regional studies have showed that dust is the major contributor of water-soluble Ca onto soil profiles, regulating the accumulation of secondary calcite in natural environments^14,45^.

The salt fraction of the highly managed pecan soils has a larger range of ^87^Sr/^86^Sr ratios (0.70922–0.71039 for Pecan_Fine (n = 29); 0.70900–0.71022 for Pecan_Coarse (n = 20)) than the pedogenic carbonate fraction (0.70965–0.71002 for Pecan_Fine (n = 28); 0.70979–0.71020 for Pecan_Coarse (n = 13)) (Fig. 4). The less managed alfalfa soils (Alfalfa_Fine_D) have ^87^Sr/^86^Sr ratios of 0.71013–0.71033 (n = 7) for salt fraction and 0.71005–0.71012 (n = 7) for pedogenic carbonate (Fig. 4). Both the Rio Grande river water (IRW_RG) and local groundwaters (IRW_GW) are used for irrigation at the pecan orchard but only Rio Grande water was used at the alfalfa site. Their ^87^Sr/^86^Sr ratios are similar (0.7099 to 0.7105, and 0.7096 to 0.7100, respectively; Appendix Table 2), and fall within the typical range of Sr isotope ratios in Rio Grande measured at other locations near El Paso during different seasons (0.7089 to 0.7150)^33^. The similarity of Sr isotope ratios between pecan and alfalfa soils to irrigation waters and their difference to the natural site strongly suggest that irrigation dominates Ca inputs onto agricultural soils. This is also in agreement with mass balance of soluble Ca from a previous study at the same site^12,14^: the irrigation water adds ~ 130 g Ca^2+^ m^−2^ yr^−1^, much higher than the fertilizers and other amendments (~ 30 g m^−2^ yr^−1^) or atmospheric deposition in the form of rain and dust (~ 0.1 g m^−2^ yr^−1^).

The salt fraction of shallow soils at the Pecan_Coarse and Pecan_Fine sites has slightly different Sr isotope ratios from that of deep soils or alfalfa soils or even their pedogenic carbonate fraction, probably due to Ca contribution from fertilizers and soil amendments (e.g., gypsum to lower soil sodicity) or groundwaters. Indeed, groundwater used for irrigation has slightly lower ^87^Sr/^86^Sr ratios than the Rio Grande river and is only applied at the pecan orchard. More likely the application of soil amendments and fertilizers has loaded soluble Ca-bearing salts at the ground surface of the pecan orchard and driven the Sr isotopes to lower values.

#### C isotope ratios of natural versus anthropogenic pedogenic carbonates

The bulk carbonate in soils is either formed naturally or induced by soil cultivation, and these two endmembers can be differentiated using carbon isotope ratios. Carbon in natural pedogenic carbonates could be sourced from atmospheric CO_2_, soil respired CO_2_ and primary carbonate bedrock^2,46–49^. Typically, soil CO_2_ is a large carbon reservoir and precipitation of secondary calcite is gradual, making soils an open system where pedogenic carbonate formation occurs at isotopic equilibrium with soil-respired CO_2_. Such relationships have been commonly documented with modern vegetation, soil CO_2_ and pedogenic calcite in arid to sub-humid environments^2,48–50^.

**Figure 5:**
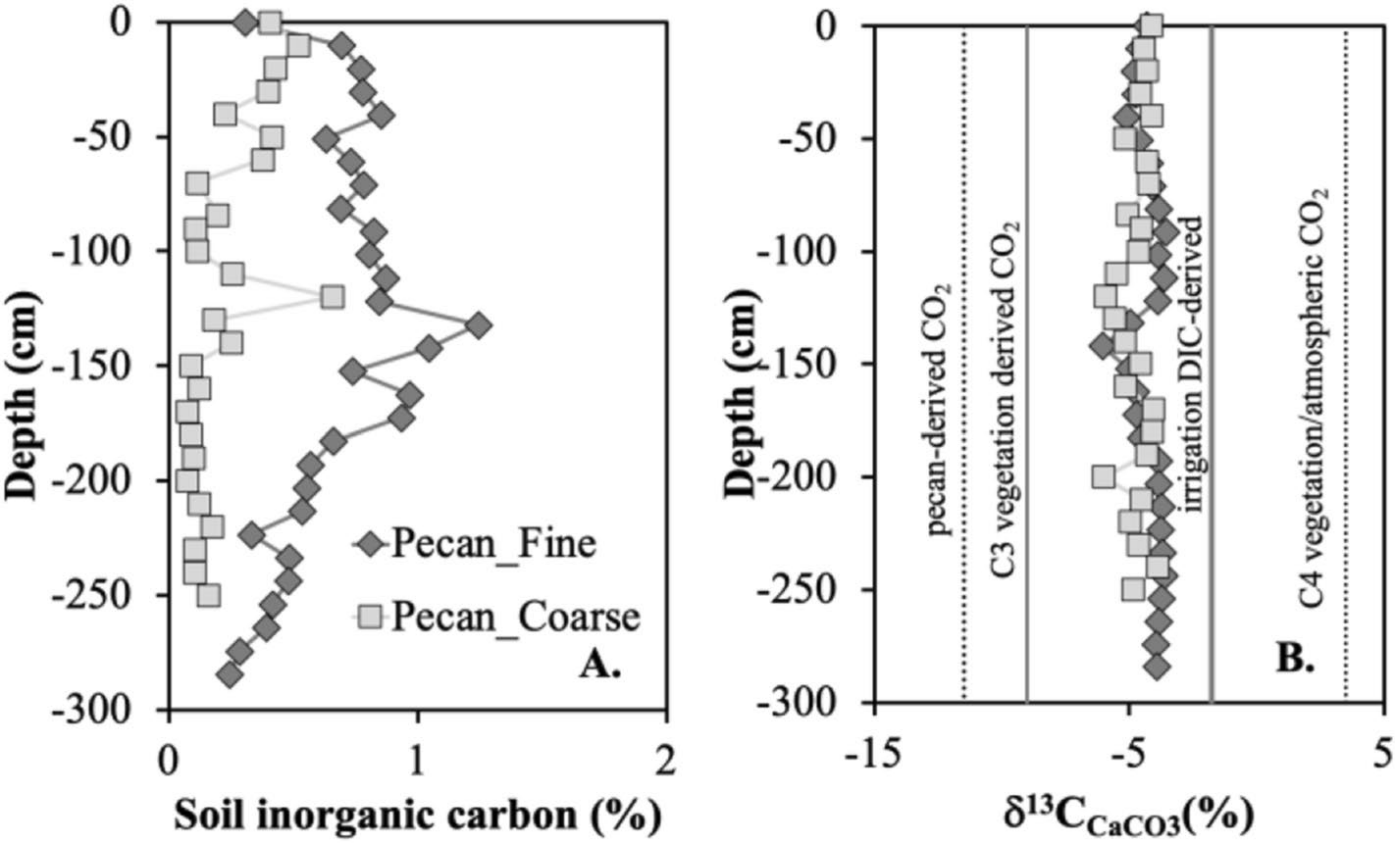
Depth profiles of soil inorganic carbon concentrations (**A**) and carbon isotopes (δ^13^C_CaCO3_) (**B**) at the Pecan_Fine and Pecan_Coarse sites. Vertical dotted in (**B**) are δ^13^C_CaCO3_ values of natural pedogenic carbonates at equilibrium with natural C3 or C4 types of vegetation. The δ^13^C_CaCO3_ at equilibrium with atmospheric CO_2_ overlaps with that of typical C4 vegetation. The solid lines are δ^13^C_CaCO3_ of anthropogenic pedogenic carbonates at equilibrium with DIC in irrigation water and with organic matter of the pecan trees.

We modeled the *δ*^13^C_CaCO3_ of natural pedogenic carbonates at equilibrium with soil CO_2_ derived from C3- and C4-type vegetation, respectively. The *δ*^13^C_SOC_ of − 27‰ was used for a typical C3 vegetation (range of − 30 to − 24‰), and − 12‰ for a typical C4 vegetation (range of − 16 to − 10‰)^42,46,47,51–57^. The soil respired CO_2_ is typically not fractionated relative to its organic matter sources, but soil CO_2_ could be enriched in ^13^C by a maximum of 4.4‰, due to fractionation via CO_2_ diffusion and mixing with atmosphere^39,41,58^. For this exercise, 4.4‰ was assumed. The equilibrium fractionation factors between soil gas CO_2_, dissolved inorganic carbon species, and calcite were calculated at 20 °C^44^, the mean annual air temperature for the region. Based on these calculations, naturally formed pedogenic carbonates with C3 and C4 types of vegetation would have *δ*^13^C_CaCO3_ values around − 11.5‰ and 3.5‰, respectively (Fig. 5B). If pedogenic carbonate precipitates at equilibrium with atmospheric CO_2_ (*δ*^13^C_CO2_ at − 8.9‰^43^), the *δ*^13^C_CaCO3_ values would be ~ 2.2‰ (Fig. 5B), overlapping with C4 vegetation.

Alternatively, carbon isotope ratios of pedogenic carbonate in irrigated soils may be controlled by *δ*^13^C_DIC_ of irrigation water, as shown in Reaction (1) in addition to soil respired CO_2_ derived from pecan trees (Fig. 1). According to the Rayleigh fractionation model in Fig. 2, irrigation DIC-induced carbonate has distinct *δ*^13^C_CaCO3_ (− 1.75‰)^44^. The pedogenic carbonate precipitated through DIC from pecan-derived and soil respired CO_2_ will be at equilibrium with *δ*^13^C_CO2_ (Fig. 1), and its *δ*^13^C_CaCO3_ would be ~ − 9‰ on average (Fig. 5B). This is plotted close to the isotope composition of pedogenic carbonate formed naturally in equilibrium with C3 vegetation derived CO_2_ since pecan is C-3 vegetation.

Soil inorganic carbon (SIC) contents are higher at the Pecan_Fine site (0.24–1.24 wt%) than those at Pecan_Coarse site (0.07–0.66 wt%) and for each soil profile, SIC peaks around the soil depth of 120 cm, above the finest soil texture (Fig. 5A). Soils at the Pecan_Fine site are finer than those at the Pecan_Coarse site, leading to lower permeability, more ponded water, higher evaporation, less leaching and more salt buildup including secondary calcite^14^. With irrigation and growth of cotton plants or pecan trees, soil gas pCO_2_ and soil water DIC in modern soils are much higher than those in natural and pre-cultivation soils, and thus carbon in pedogenic carbonates of bulk soils is dominated by anthropogenic sources (Fig. 5B). The carbon isotope compositions of bulk pedogenic carbonates from the two pecan profiles are similar ranging from − 3.7 to − 6.0‰ and fall in the mixing zone of these two endmembers.

### Improve our capacity to quantify and model future changes in pedogenic carbonate accumulation rates and abiotic CO_2_ emission fluxes

The uncertainty on the relative contribution of old and natural versus irrigation-derived young pedogenic carbonates, or relative contribution of soil respired, atmospheric and abiotic CO_2_ (X_A_ or X_B_ in Eq. (8)) relies on several assumptions. First, this calculation assumes a uniform C or Sr isotope composition for each endmember. For irrigation, this depends on proportion of different water sources and the variation of their C or Sr isotope ratios. Regional groundwater typically has much longer age and mixing time with relative constant ^13^C_DIC_ or ^87^Sr/^86^Sr; however, Rio Grande, the river water has seasonal fluctuation as observed by just several C and Sr isotope data reported in this study (Appendix Table 2). Future work is needed to capture all irrigation events for at least one year. It is noted that for the pecan site, farmers switched to pecan production after 60 years of cotton. This transition would have led to a very small shift in the C isotopes of soil respired CO_2_, as both cotton and pecan are C3 crops and have similar carbon assimilation pathways^59,60^.

Second, Eq. (8) assumes two-endmember mixing only, natural and anthropogenic sources. Both soil profiles at the pecan orchard showed less radiogenic Sr isotope values near the ground surface, for both fractions of salts and pedogenic carbonates, but soils at the alfalfa site had nearly constant Sr isotope ratios with depth (Fig. 4). Such a difference might be due to application of soil amendments and fertilizers at the pecan orchard but not at the alfalfa field^14^. Amendments-derived Ca flux is small but could concentrate near surface and shift soil Sr isotopes, especially in the winter months when the soils were collected and before the irrigation dissolved and leached these soluble salts to deeper soils. Only one of the soil treatments reported^14^, urea, was measured for the ^87^Sr/^86^Sr ratio and it was higher than all soil leachates (0.7106). Future Sr isotope measurements on other fertilizers and amendments will better constrain the isotope shift at shallow soils and quantify the Ca inputs.

The accumulation rates of salts and pedogenic carbonate are expected to increase over time in irrigated fields of the Rio Grande valley, threatening agricultural sustainability and increasing its carbon footprint. Soil texture varies with depth and location due to different types of floodplain sediments that deposited several thousands of years ago^13^ and reduced infiltration near the fine-texture soils allows faster salt accumulation rates^14^. As a consequence, the accumulation of secondary salts and carbonate has clogged pores, leading to less leaching and more salt buildup. Such a positive feedback thus projects faster pedogenic carbonate precipitation and more abiotic CO_2_ emission in the future. This accumulation of pedogenic carbonate can only be reversed if land management practice changes and irrigation with much fresher water leads to dissolution of existing carbonate.

From the perspective of the irrigation water chemistry, the total dissolved solids (TDS) of the groundwater from the alluvial aquifers or the Hueco Bolson and Mesilla Bolson are variable between those of freshwater and brackish water, but typically higher than that of the Rio Grande river^14,61,62^. If the Rio Grande river is less available due to reduced snowfall in Colorado, the headwater region^63,64^, then more groundwater is used for irrigation, accelerating salt loading and pedogenic carbonate formation rates. The salt loading rates through irrigation are proportional to amount of water applied and their chemistry. Flood irrigation is widely used along the Rio Grande valley and other regions where fields inundate with water without any infrastructure^65,66^. The evaporative water loss during the irrigation season is problematic for freshwater-limited drylands. In order to slow down the salt buildup process, much more effective irrigation methods should be used to require less water and load less salt.

### Is pedogenic CO_2_ efflux important to global C cycling?

Pedogenic carbonate prevails in dryland systems from subhumid to subarid climate conditions^2,67,68^, and is estimated to contain 700 to 940 Pg of carbon (1Pg = 10^15^ g), which is similar in size to the atmospheric carbon pool and about two-thirds of soil organic carbon pools^2,69–71^. However, pedogenic carbonate formation in natural drylands is slow, limited by soluble Ca inputs and with almost undetectable abiotic CO_2_ in field studies^72^.

Our data indicate that continuous supply of Ca- and DIC-rich irrigation waters has accelerated pedogenic carbonate formation and promoted the subsequent release of abiotic CO_2_ in dryland agricultural fields. It is critical to assess if this abiotic CO_2_ is a major component in the carbon cycling. One modeling effort suggested ~ 2.2 Tg C yr^−1^ (1Tg = 10^12^ g) were emitted to the atmosphere from ~ 16 million ha (or 0.16 million km^2^) of irrigated fields in the western U.S.^73^. This is equivalent to an emission of abiotic CO_2_ to the atmosphere and accumulation of pedogenic carbonate both at 14 g C m^−2^ yr^−1^, one to two orders of magnitude higher than these fluxes in natural settings^53,74^. Pedogenic carbonate accumulation rates were quantified at ~ 1 gC m^−2^ yr^−1^ (or 9 gCaCO_3_ m^−2^ yr^−1^) in the alfalfa site, but these have been underestimated due to large error bars in U-disequilibrium dating methods^13^. We estimated abiotic CO_2_ emission at 48 gC m^−2^ yr^−1^ through irrigation and if this is true for all other agricultural drylands, then a potential total of *ca.* 48 gC m^−2^ yr^−1^ * 0.4 million km^2^ = 19 Tg C yr^−1^ is released as abiotic CO_2_ following irrigation. This value is 9 times the flux estimated for southwestern US alone^73^.

Soil salinization has challenged food production, affecting approximately 20% of irrigated lands globally^5^. Since calcite has lower solubility than evaporite salts, soils with elevated salinity should also be characterized by accumulation of pedogenic carbonates. Combined, these findings suggest dryland agriculture has the potential to significantly alter land–atmosphere CO_2_ flux over a large area of the Earth’s surface.

Two important factors along the Rio Grande valley are soil texture that controls infiltration rates and salt leaching versus accumulation (Pecan_Fine vs. Pecan_Coarse; Alfalfa_Fine vs. Alfalfa_Coarse), and irrigation chemistry and intensity, that dictate the maximum possible loading of salts (river water vs. groundwater)^12,14^. These are similar to the chemistry of irrigation water and hydrological conditions emphasized in previous models^73^, and so, abiotic CO_2_ efflux rates associated with pedogenic carbonate accumulation are likely to vary significantly among agricultural settings.

## Conclusion

To summarize, both irrigation waters and fertilizers/soil amendments load DIC and Ca^2+^ and promote the accumulation of pedogenic carbonate. This has not only increased SIC contents, but also shifted carbon and strontium isotope ratios of pedogenic carbonate in the agricultural fields. Accumulation rates of pedogenic carbonates are greatly elevated due to flood irrigation even if soil cultivation with flood irrigation has only occurred for the last 100 years, and even in soils that have naturally accumulated secondary calcite over thousands of years since the deposits of these flood plains of the Rio Grande.

Evaporite salts and pedogenic carbonates in agricultural soils have ^87^Sr/^86^Sr signatures similar to irrigation waters (Rio Grande and local groundwater), with slight modification from soil amendments and fertilizers near the soil surface. In contrast, different ^87^Sr/^86^Sr ratios in salt vs. pedogenic carbonates are observed in natural soils, indicative of dust-derived Ca. Similarly, carbon isotopes of pedogenic carbonate in these soils fall within the range of two endmembers, soil respired CO_2_ from C3 crop and DIC in the irrigation water. This study has clearly identified that the pedogenic carbonates in agricultural soils along the Rio Grande valley are predominantly formed by loading of dissolved inorganic carbon and dissolved calcium through flood-irrigation. This is in agreement with a mass balance calculation at the same sites that showed irrigation loaded approximately 350 g Ca^2+^ m^−2^ yr^−1^, much higher than natural sources as rain or dust^14^.

Because of the coupling between calcite production and CO_2_ release in Reaction (1), we conclude that calcite-derived CO_2_ is an important CO_2_ source in agricultural soils. Indeed, Keeling plots of soil gas samples in a pecan orchard revealed the contribution of such an abiotic CO_2_ endmember. To the best of our knowledge, irrigation derived CO_2_ is observed for the first time in our field studies. This adds to the literature that mineral–water interaction has the potential to modify CO_2_ efflux and our estimates of soil respired rates.

Based on this study and previous works, soil texture, irrigation intensity, and water chemistry are identified as the dominant controls on accumulation rates of pedogenic carbonate and emission fluxes of abiotic CO_2_. If land management practices are not changed to decrease salt loading by irrigation, negative consequences are expected, where secondary minerals will clog pores and reduce infiltration, leading to elevated sodicity and salinity and accelerating calcite precipitation rates and subsequent CO_2_ emissions. Future climate projections will likely amplify pressures in this region: reduced river flow in the future may promote use of more concentrated groundwater, and hot/dry summers may lead to more evaporative water loss. Additional studies are needed to identify major variables that control the pedogenic carbonate accumulation rates and the accompanied abiotic CO_2_ emission, to investigate the temporal and spatial variability within an agricultural field, and more importantly scale up from the Rio Grande valley to other dryland regions.

## Supplementary Information

Supplementary Information.
