# Supplementary material for: Dryland irrigation increases accumulation rates of pedogenic carbonate and releases soil abiotic CO2
Source: Sci Rep. 2022 Jan 10;12:464. doi: 10.1038/s41598-021-04226-3 (PMC8748926; doi:10.1038/s41598-021-04226-3)
Supplement: Supplementary file 1 — Supplementary Information. [file 41598_2021_4226_MOESM1_ESM.pdf]

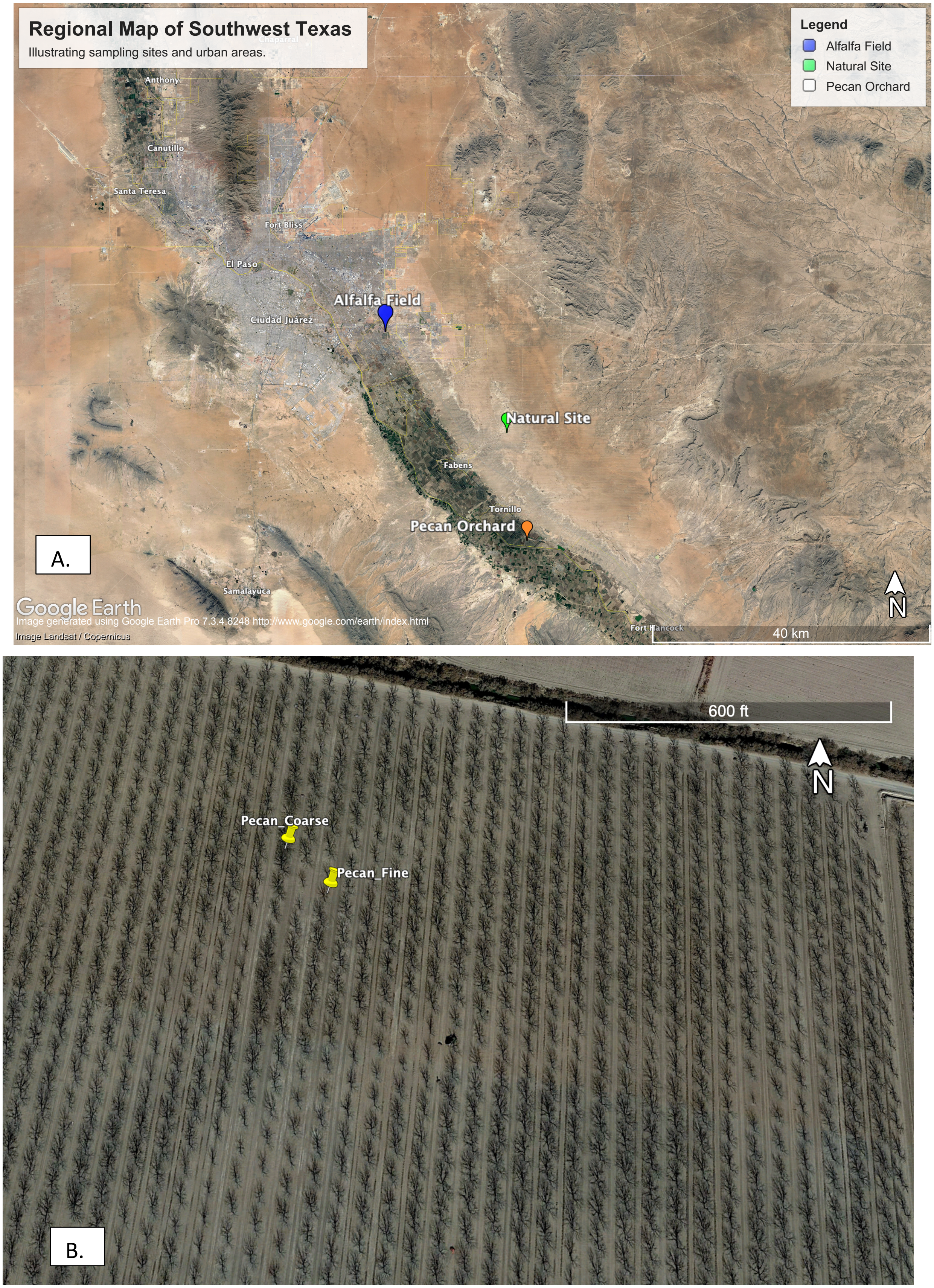
Regional Map of Southwest Texas
Illustrating sampling sites and urban areas.
Legend
Alfalfa Field
Natural Site
Pecan Orchard
Anthony
Canutillo
Santa Teresa
Fort Bliss
El Paso
Alfalfa Field
Ciudad Juárez
Natural Site
Fabens
Tornillo
Pecan Orchard
A.
Samalayuca
Google Earth
Image generated using Google Earth Pro 7.3.4.8248 http://www.google.com/earth/index.html
Image Landsat / Copernicus
Fort Hancock
40 km
N
600 ft
N
Pecan_Coarse
Pecan_Fine
B.

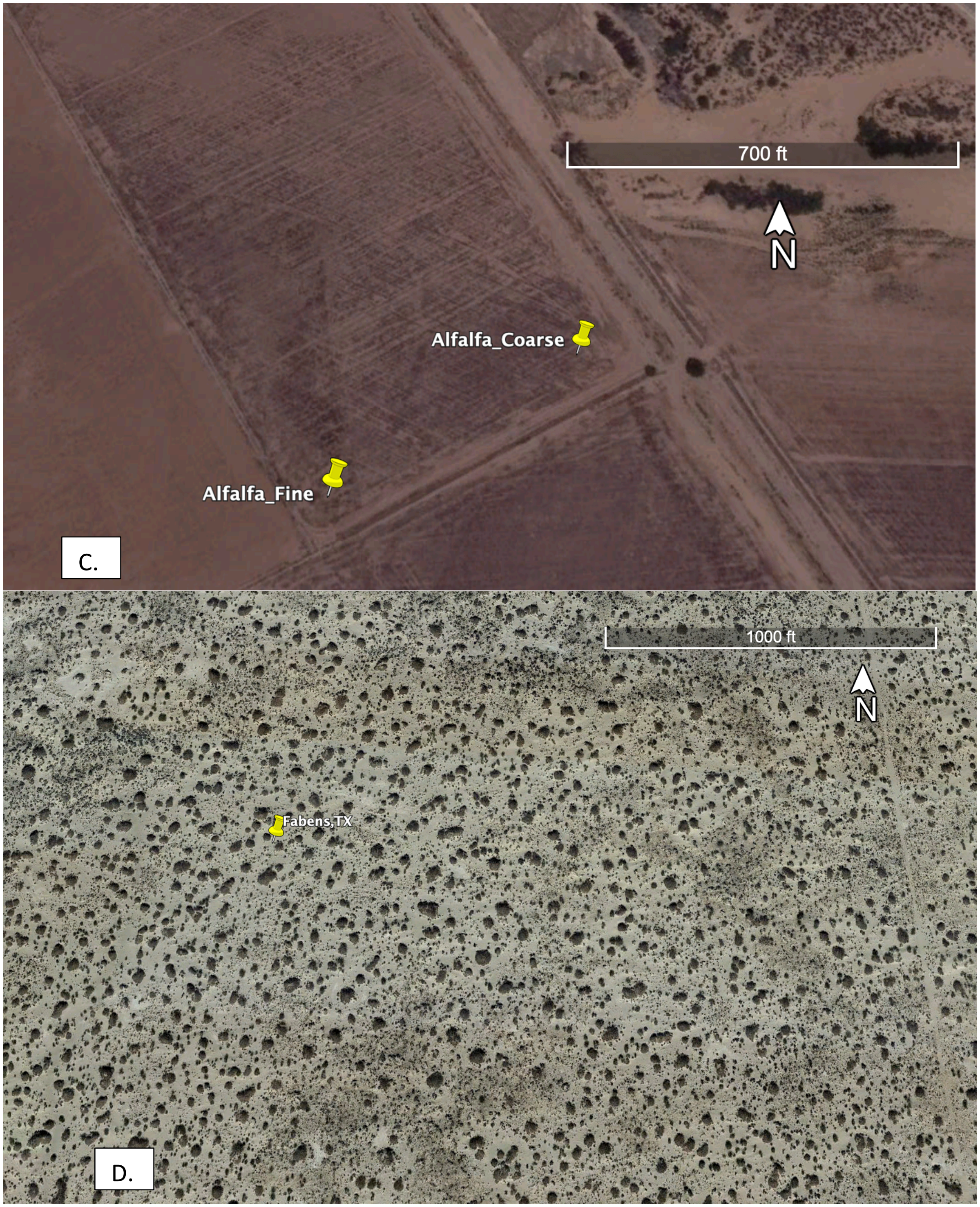

Figure S1: Three study sites are located in western Texas and southern New Mexico in Southwestern United States (A). The Rio Grande river flows through El Paso, Texas and becomes the international boarder. The river water is diverted through canals and used for irrigation in farms along the Rio Grande valley, including two agricultural sites in this study: the pecan orchard (B) and the alfalfa farm (C). In addition, a natural site is included at Fabens, Texas (D). Image are generated using Google Earth Pro 7.3.4.8248 on 11/2/2021 via http://www.google.com/earth/index.html

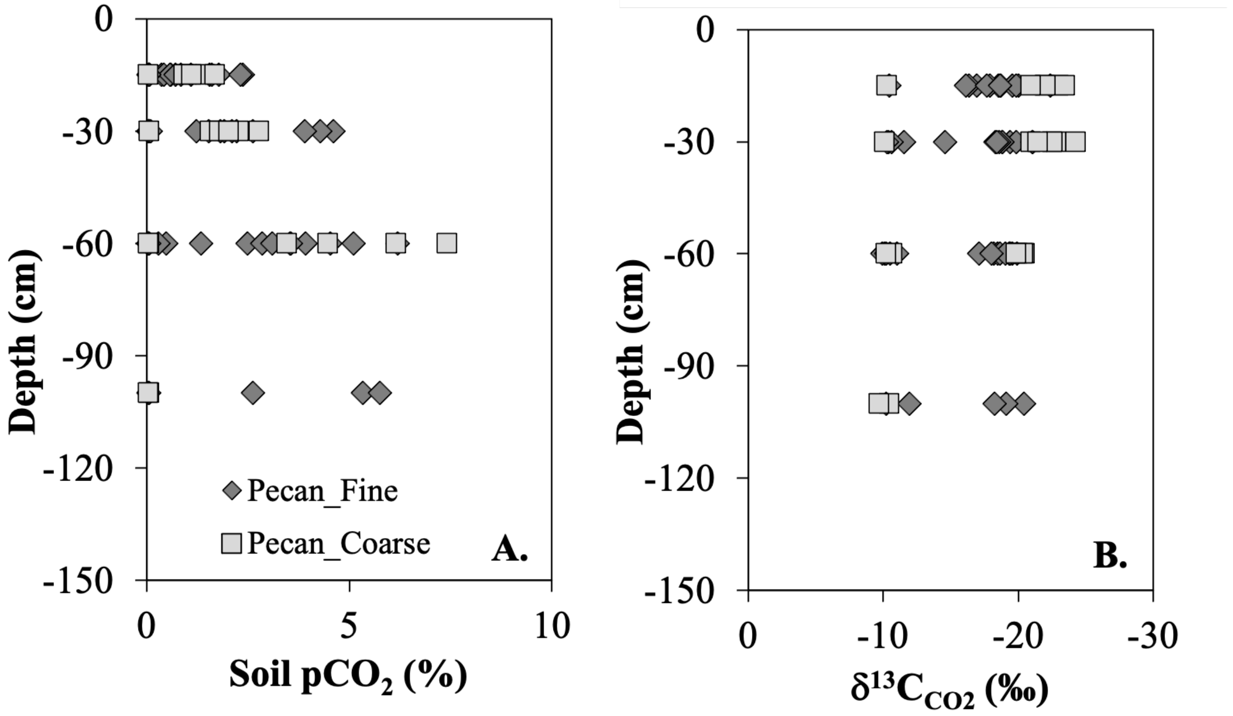

Figure S2: Depth profiles of soil gas CO_2 concentrations (A) and carbon isotopes ($\delta^{13}C_{CO2}$, B) at the Pecan_Fine and Pecan_Coarse sites. Each soil gas sampler was sampled multiple times, showing seasonal variation in pCO_2 and $\delta^{13}C_{CO2}$.

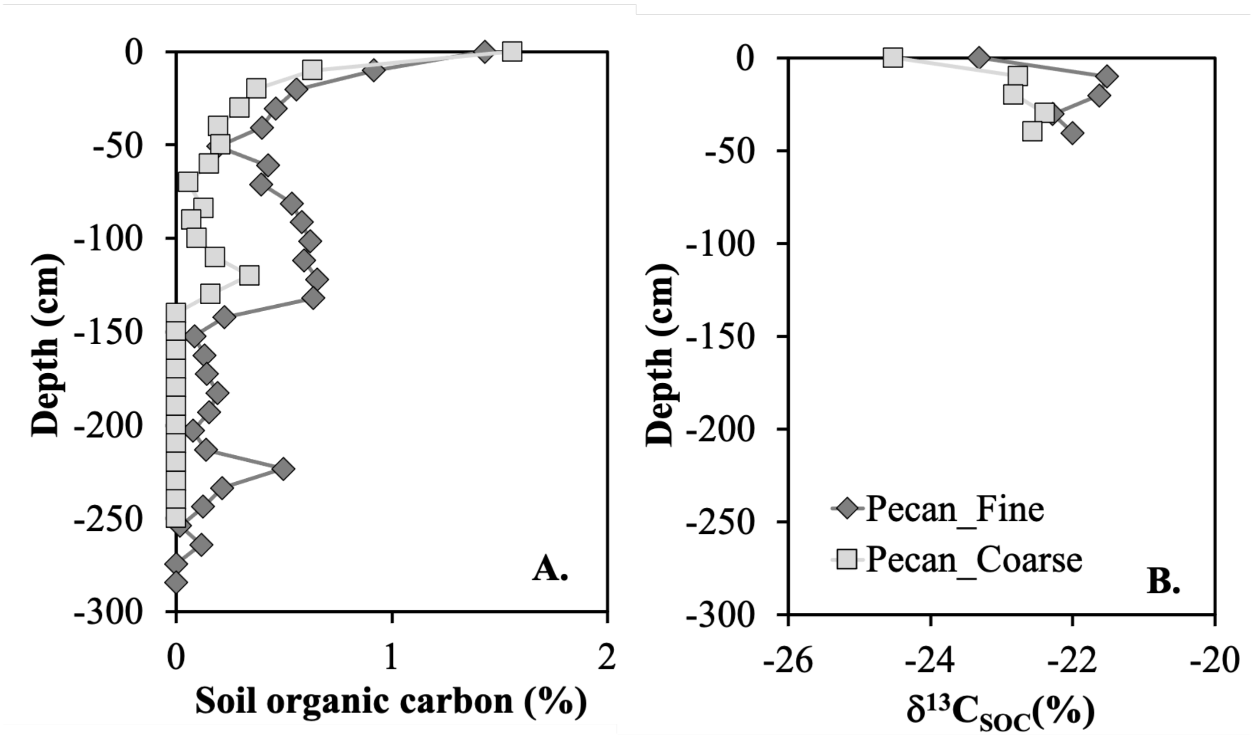

Figure S3: Depth profiles of soil organic carbon concentrations (A) and carbon isotopes ($\delta^{13}C_{SOC}$, B) at the Pecan_Fine and Pecan_Coarse sites.

Appendix Table 1: Concentrations and C isotope composition of soil gas CO_2 from two soil profiles

Collection Date	Depth (cm)	pCO_2 (%)	pCO_2 (ppm)	$\delta^{13}C_{CO2}$ ‰	Collection Date	Depth (cm)	pCO_2 (%)	pCO_2 (ppm)	$\delta^{13}C_{CO2}$ ‰
**Pecan_Fine**					**Pecan_Coarse**				
5/2/14	15	0.36	3580	-18.7	5/1/15	15	0.05	506	-10.3
5/19/14	15	0.43	4323	-18.5	5/21/15	15	0.99	9924	-23.0
6/5/14	15	1.78	17775	-16.9	6/17/15	15	1.46	14571	-23.4
6/25/14	15	0.84	8358	-19.6	6/30/15	15	0.93	9259	-22.0
7/15/14	15	0.60	5983	-16.4	7/10/15	15	1.68	16770	-22.1
7/24/14	15	0.06	560		7/19/15	15	1.11	11096	-21.0
7/31/14	15	0.05	522		5/1/15	30	0.05	517	-10.1
8/14/14	15	0.05	494	-10.5	5/21/15	30	1.57	15743	-22.9
9/1/14	15	1.56	15552	-17.9	6/17/15	30	2.28	22812	-22.5
10/9/14	15	0.57	5711	-16.1	6/30/15	30	1.79	17917	-20.9
10/23/14	15	0.06	557		7/10/15	30	2.77	27746	-21.4
5/1/15	15	0.05	542		7/19/15	30	2.03	20332	-24.3
5/21/15	15	1.09	10948	-22.4	5/1/15	60	0.05	523	-10.7
6/17/15	15	2.37	23737	-20.0	5/21/15	60	3.46	34587	-20.0
6/30/15	15	0.70	7033	-17.7	6/17/15	60	4.47	44738	-20.5
7/10/15	15	2.31	23137	-19.8	6/30/15	60	0.05	498	-10.2
7/19/15	15	1.62	16165	-18.7	7/10/15	60	7.41	74073	-20.4
5/2/14	30	0.06	563		7/19/15	60	6.15	61453	-19.9
6/5/14	30	4.61	46091	-19.4	5/1/15	100	0.05	515	-10.4
6/25/14	30	2.10	20984	-18.3	6/30/15	100	0.05	481	-9.7
7/15/14	30	1.82	18248	-14.6					
7/24/14	30	1.91	19081	-19.8					
7/31/14	30	0.05	514	-11.6					
8/14/14	30	0.05	474	-10.4					
9/1/14	30	4.27	42744	-18.8					
10/9/14	30	1.52	15223	-18.8					
10/23/14	30	0.05	493	-10.6					
5/1/15	30	0.05	517	-10.3					
5/21/15	30	2.21	22147	-21.1					
6/17/15	30	3.90	38994	-21.1					
6/30/15	30	1.23	12255	-18.6					
7/10/15	30	0.10	1025						
7/19/15	30	2.62	26160	-18.4					
5/2/14	60	2.49	24851	-19.6					
5/19/14	60	3.55	35452						
6/5/14	60	0.48	4817	-19.4					
6/25/14	60	3.91	39093	-17.1					
7/15/14	60	0.30	2964	-18.2					
7/24/14	60	1.35	13484	-19.9					
7/31/14	60	0.05	488	-10.1					
8/14/14	60	0.05	488	-9.9					
9/1/14	60	6.20	62002	-18.5					
10/9/14	60	2.85	28464	-18.6					
10/23/14	60	0.05	507	-11.0					
5/1/15	60	0.05	501	-10.2					
5/21/15	60	3.10	30995	-19.4					
6/17/15	60	4.53	45312	-19.4					
6/30/15	60	0.05	515	-10.5					
7/10/15	60	5.10	50971	-19.0					
7/19/15	60	3.55	35512	-18.0					
5/1/15	100	0.05	496	-10.2					
5/21/15	100	2.63	26285	-20.4					
6/30/15	100	0.05	512	-12.0					
7/10/15	100	5.33	53279	-19.1					
7/19/15	100	5.75	57482	-18.2					

Appendix Table 2: Elemental and isotopic data for the irrigation water samples collected for this study

Name	Collection Date	Sample Source	pH	EC (dS/m)	alkalinity (meq/L)	$\delta^{13}C_{DIC}$ ‰	$^{87}Sr/^{86}Sr$	2SE	Na^+ ppm	Ca^{2+} ppm	Mg^{2+} ppm	K^+ ppm	Cl^- ppm	SO_4^{2-} ppm	calcite Saturation index
IRW_GW	4/17/15	WELL1	7.38	3.77	5.3	-10.5	0.70963	0.00001	474	310	65	61	887	658	0.6
IRW_GW	4/17/15	WELL2	7.25	4.26	6.8	-12.2	0.70995	0.00001	597	266	60	57	741	1051	0.4
IRW_RG	5/15/15	Rio Grande	7.70	1.87	3.5	-10.4	0.71007	0.00001	325	43	13	67	340	333	0.0
IRW_RG	6/11/15	Rio Grande	8.10	1.02	3.7	-6.6	0.71047	0.00001	126	39	11	60	118	192	0.5
IRW_RG	6/30/15	Rio Grande	8.00	0.90	3.5	-6.3	0.71031	0.00002	115	36	10	57	91	159	0.3
IRW_RG	8/11/15	Rio Grande	7.91	0.88	3.7	-6.5	0.71048	0.00001	102	56	8	9	93	155	0.3
IRW_RG	8/30/15	Rio Grande	7.90	0.96	3.4	-7.2			56	98	16	12	96	169	0.4
IRW_RG	9/22/15	Rio Grande				-6.8	0.71013	0.00001	28	92	15	11	103	170	

IRW_GW and IRW_RG: irrigation water, from groundwater and Rio Grande.

Appendix Table 3: Elemental and isotope compositions in soil samples

Site	IGSN	Depth	soil inorganic carbon	soil organic carbon	$\delta^{13}C_{CaCO3}$	$\delta^{13}C_{SOC}$	$^{87}Sr/^{86}Sr$	2SE	$^{87}Sr/^{86}Sr$	2SE
		(cm)	wt %	wt %	‰	‰	Salts/water leachable		carbonate/acid leachable	
**Pecan_Fine**										
P3_0_4	IEELP0001	0	0.31	1.43	-4.3	-23.3	0.70946	0.00005	0.70965	0.00002
P3_4_8	IEELP0002	10	0.69	0.92	-4.6	-21.5	0.70960	0.00002	0.70975	0.00002
P3_8_12	IEELP0003	20	0.77	0.56	-4.8	-21.6	0.70980	0.00010	0.70984	0.00001
P3_12_16	IEELP0004	30	0.78	0.46	-4.7	-22.3	0.71005	0.00004	0.71001	0.00002
P3_16_20	IEELP0005	41	0.85	0.40	-5.1	-22.0	0.71008	0.00002	0.71002	0.00002
P3_20-24	IEELP0006	51	0.63	0.19	-4.6		0.70922	0.00049	0.71008	0.00003
P3_24_28	IEELP0007	61	0.73	0.43	-4.2		0.70955	0.00029	0.70998	0.00003
P3_28_32	IEELP0008	71	0.78	0.39	-4.1		0.71005	0.00095	0.70998	0.00002
P3_32_36	IEELP0009	81	0.69	0.54	-3.9		0.71026	0.00002	0.70999	0.00002
P3_36_40	IEELP000A	91	0.83	0.58	-3.6		0.71039	0.00008	0.71001	0.00002
P3_40_44	IEELP000B	102	0.80	0.62	-3.9		0.71010	0.00013	0.71004	0.00006
P3_44_48	IEELP000C	112	0.87	0.59	-3.6		0.71027	0.00010	0.71016	0.00002
P3_48_52	IEELP000D	122	0.84	0.65	-3.9		0.71021	0.00002	0.71019	0.00002
P3_52_56	IEELP000E	132	1.24	0.63	-5.0		0.71025	0.00002	0.71023	0.00002
P3_56_60	IEELP000F	142	1.05	0.22	-6.0		0.71015	0.00002	0.71028	0.00002
P3_60_64	IEELP000G	152	0.74	0.08	-5.0		0.71012	0.00002	0.71010	0.00002
P3_64_68	IEELP000H	163	0.97	0.13	-4.7		0.71024	0.00002	0.71016	0.00002
P3_68_72	IEELP000I	173	0.93	0.14	-4.7		0.71021	0.00002	0.71013	0.00002
P3_72_76	IEELP000J	183	0.66	0.19	-4.5		0.71021	0.00002	0.71007	0.00002
P3_76_80	IEELP000K	193	0.57	0.15	-3.9		0.71025	0.00001	0.71005	0.00002
P3_80_84	IEELP000L	203	0.55	0.08	-3.9		0.71023	0.00002	0.71003	0.00003
P3_84_88	IEELP000M	213	0.53	0.14	-3.7		0.71026	0.00001	0.71003	0.00003
P3_88_92	IEELP000N	224	0.33	0.49	-3.8		0.71020	0.00002	0.70996	0.00004
P3_92_96	IEELP000O	234	0.48	0.21	-3.7		0.70992	0.00001	0.71006	0.00002
P3_96_100	IEELP000P	244	0.48	0.12	-3.7		0.71027	0.00002	0.70997	0.00003
P3_100_104	IEELP000Q	254	0.42	0.02	-3.7		0.71020	0.00002	0.70997	0.00002
P3_104_108	IEELP000R	264	0.39	0.12	-3.9		0.71000	0.00002	0.70994	0.00002
P3_108_112	IEELP000S	274	0.28	0	-4.0		0.71024	0.00002	0.70997	0.00002
P3_112_116	IEELP000T	284	0.24	0	-3.9		0.71019	0.00002		
**Pecan_Coarse**										
P4_0_10	IEELP000U	0	0.41	1.56	-4.2	-24.5	0.70950	0.00033		
P4_10_20	IEELP000V	10	0.52	0.63	-4.4	-22.8			0.70979	0.00002
P4_20_30	IEELP000W	20	0.43	0.37	-4.3	-22.8	0.70932	0.00035	0.70987	0.00002
P4_30_40	IEELP000X	30	0.40	0.29	-4.6	-22.4	0.70901	0.00091		
P4_40_50	IEELP000Y	40	0.22	0.19	-4.2	-22.6	0.70951	0.00045	0.70992	0.00002
P4_50_60	IEELP000Z	50	0.42	0.21	-5.2		0.71003	0.00009		
P4_60_70	IEELP0010	60	0.38	0.15	-4.3		0.70988	0.00018	0.71000	0.00002
P4_70_84	IEELP0011	70	0.11	0.06	-4.2		0.70972	0.00033		
P4_84_90	IEELP0012	84	0.20	0.13	-5.1		0.71022	0.00022	0.71001	0.00003
P4_90_100	IEELP0013	90	0.11	0.07	-4.5		0.70996	0.00011	0.71002	0.00002
P4_100_110	IEELP0014	100	0.11	0.10	-4.7		0.71000	0.00014		
P4_110_120	IEELP0015	110	0.26	0.18	-5.5		0.71014	0.00005	0.71007	0.00002
P4_120_130	IEELP0016	120	0.66	0.34	-5.9		0.71019	0.00004		
P4_130_140	IEELP0017	130	0.18	0.16	-5.6		0.70987	0.00023	0.71009	0.00002
P4_140_150	IEELP0018	140	0.25	0	-5.2				0.70999	0.00006
P4_150_160	IEELP0019	150	0.09	0	-4.6		0.71013	0.00011	0.71014	0.00002
P4_160_170	IEELP001A	160	0.12	0	-5.1		0.70900	0.00065		
P4_170_180	IEELP001B	170	0.07	0	-4.1		0.71020	0.00014		
P4_180_190	IEELP001C	180	0.09	0	-4.1		0.70944	0.00053	0.71008	0.00002
P4_190_200	IEELP001D	190	0.10	0	-4.3					
P4_200_210	IEELP001E	200	0.07	0	-6.0		0.70983	0.00017	0.71016	0.00002
P4_210_220	IEELP001F	210	0.12	0	-4.5				0.71020	0.00003
P4_220_230	IEELP001G	220	0.17	0	-5.0		0.70993	0.00009		
P4_230_240	IEELP001H	230	0.10	0	-4.7					
P4_240_250	IEELP001I	240	0.10	0	-3.9		0.70990	0.00037		
P4_250_260	IEELP001J	250	0.16	0	-4.9					
**Alfalfa_Fine1B**										
0-10		0					0.71031	0.00001	0.71011	0.00001
10--20		10					0.71030	0.00001	0.71011	0.00001
20-30		20					0.71033	0.00001	0.71012	0.00001
30-40		30					0.71031	0.00001	0.71011	0.00001
40-50		40					0.71028	0.00001	0.71011	0.00001

Appendix Table 3: Elemental and isotope compositions in soil samples

Site	IGSN	Depth (cm)	soil inorganic carbon wt %	soil organic carbon wt %	$\delta^{13}C_{CaCO3}$ ‰	$\delta^{13}C_{SOC}$ ‰	$^{87}Sr/^{86}Sr$ Salts/water leachable	2SE	$^{87}Sr/^{86}Sr$ carbonate/acid leachable	2SE
50-60		50					0.71013	0.00002	0.71008	0.00001
60-70		60					0.71025	0.00010	0.71005	0.00001
**Natural Fabens**										
0-20	IEELP002V	0					0.70901	0.00019	0.70940	0.00010
20-40	IEELP002W	20					0.70941	0.00007	0.70938	0.00002
40-60	IEELP002X	40					0.70928	0.00002	0.70940	0.00002
60-71	IEELP002Y	60					0.70931	0.00004	0.70941	0.00002
71-81	IEELP002Z	71					0.70933	0.00002	0.70940	0.00002
81-90	IEELP0030	81					0.70935	0.00009	0.70935	0.00008
90-99	IEELP0031	90					0.70934	0.00002	0.70941	0.00002
99-109	IEELP0032	99					0.70937	0.00002	0.70921	0.00010
**Dust**										
Natural Fabens							0.70914	0.00016	0.71012	0.00001
Pecan Orchard							0.70937	0.00034	0.71088	0.00002
